# Extramedullary versus intramedullary femoral alignment technique in total knee arthroplasty: a meta-analysis of randomized controlled trials

**DOI:** 10.1186/s13018-017-0582-3

**Published:** 2017-06-05

**Authors:** Qian Tang, Ping Shang, Gang Zheng, Hua-Zi Xu, Hai-Xiao Liu

**Affiliations:** 1 Department of Orthopaedic Surgery, The Second Affiliated Hospital and Yuying Children’s Hospital of Wenzhou Medical University, 109, Xueyuanxi road, 325027 Wenzhou, China; 2 Department of Rehabilitation, The Second Affiliated Hospital and Yuying Children’s Hospital of Wenzhou Medical University, 109 Xueyuanxi road, Wenzhou, 325027 China

**Keywords:** Total knee arthroplasty, Meta-analysis, Blood loss

## Abstract

**Background:**

There is no consensus whether the use of the extramedullary femoral cutting guide takes advantage over the intramedullary one in total knee arthroplasty. The aim of this study was to compare the extramedullary femoral alignment guide system with the conventional intramedullary alignment guide system for lower limb alignment, blood loss, and operative time during total knee arthroplasty.

**Methods:**

The Medline, Embase, Cochrane Library, China National Knowledge Infrastructure (CNKI), Wan Fang Chinese Periodical, Google, and reference lists of all the included studies were searched for randomized controlled trials. The following parameters were compared between the extramedullary technique and the intramedullary technique: (1) lower limb coronal alignment, (2) coronal alignment of femoral component, (3) sagittal alignment of femoral component, (4) blood loss, (5) and operation time.

**Results:**

Four randomized controlled trials consisting of 358 knees were included in our study. There was no significant difference between the extramedullary and intramedullary groups for the lower limb coronal alignment (RR = 1.20, 95%CI 0.28~5.21, n.s.), coronal alignment of femoral component (RR = 0.65, 95%CI 0.19~2.22, n.s.), and sagittal alignment of femoral component (RR = 0.73, 95%CI 0.38~1.41, n.s.). A reduced blood loss was associated with the use of the extramedullary guide (MD = −120.34, 95%CI −210.08~−30.59, *P* = 0.009). No significant difference in operation time was noted between the two groups (MD = 1.41, 95%CI −1.82~4.64, n.s.).

**Conclusions:**

Neither extramedullary nor intramedullary femoral alignment is more accurate than the other in facilitating the femoral cut in total knee arthroplasty. Use of the extramedullary guide results in less blood loss and exhibits a similar operation time as compared with the intramedullary guide.

**Electronic supplementary material:**

The online version of this article (doi:10.1186/s13018-017-0582-3) contains supplementary material, which is available to authorized users.

## Background

In total knee arthroplasty (TKA), the prosthetic placement and overall limb alignment has been demonstrated to be most influential in determining implant survival [1–4]. The ideal position for the components recreates a neutral mechanical axis. Most surgeons currently favor intramedullary (IM) alignment for its ease of use and accuracy as compared with extramedullary (EM) alignment. Previous comparisons of IM and EM femoral alignment systems have shown the former to be more accurate in performing the distal femoral cut [5–7]. In these studies, the IM alignment technique has ranged from 85 to 96% in the normal range as compared with 69 to 86% for the EM alignment technique.

However, the IM femoral alignment system does not always guarantee the accuracy of the component position in TKA. Previous studies used only the anterosuperior iliac spine as an intraoperative landmark for the EM referencing instruments [5–7]. Intraoperative visual assessment of the longitudinal femur axis and the anterosuperior iliac spine by EM rods is difficult owing to the large soft tissue cover and tourniquets. TKAs using extramedullary femoral alignment guides have been extensively studied in recent years. Extramedullary instruments using newly designed mechanical axis marker systems have provided as accuracy in reproducing a neutral distal femoral resection on the coronal and sagittal planes during TKA as standard IM instruments [8–10].

Recent advances in TKA have focused on the reduction of damage during the procedure [11–13]. One of the most invasive parts of TKA is the violation of the intramedullary femoral canal and the subsequent use of IM instruments. The use of an intramedullary guide for the femur can result in various complications such as blood loss, postoperative hypoxia, intraoperative fractures, and fat embolism [11, 14, 15], while the EM method has less morbidity in terms of blood loss because it does not invade bone marrow. In addition, the IM instrument may not be applicable when a long-stemmed femoral component implanted during a previous surgery remains or a rod cannot be inserted due to severe deformity of the femur [9].

In this study, we conducted a meta-analysis of pooled the data from relevant RCTs to evaluate whether an IM or EM femoral guide is more accurate in assuring correct femoral positioning. Moreover, the blood loss and operation time were also compared between these two techniques. Our hypothesis was that the EM femoral guide provided similar accuracy for femoral positioning and less blood loss compared with the IM femoral guide during TKAs. If the hypothesis is confirmed, the EM femoral guide may provide an alternative approach for femoral cuts and display clinical benefit for particular cases, such as a previous surgery remains and severe deformity of the femur.

## Methods

### Database retrieval

**Fig. 1:**
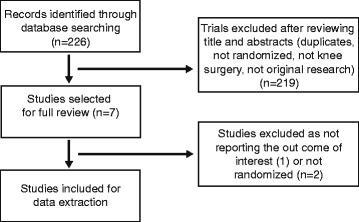
Flow chart of the study selection and inclusion process

The present study was conducted using the Preferred Reporting Items for Systematic Reviews and Meta Analyses (PRISMA) statement. We conducted this meta-analysis of all English and non-English articles identified from electronic databases including Medline, Embase, Cochrane Library, China National Knowledge Infrastructure, Wan Fang Chinese Periodical, and Google. In addition, we also manually searched for other relevant studies including those from the reference lists of all included studies. The last search was conducted on December 6, 2015. We used the following key words: arthroplasty, replacement, femoral, total knee arthroplasty, randomized, randomised, intramedullary, extramedullary, in combination with the Boolean operators AND or OR. The search strategy is presented in Fig. 1.

### Quality criteria of trials and data extraction

We included all published RCTs comparing EM guide with IM guide in patients undergoing primary TKA. Exclusion criteria comprised the following: trials with a retrospective design and trials that did not randomize patients into two relevant groups. Quality criteria included the randomization method, concealment of allocation, blinding, and intention-to-treat analysis.

For each eligible study, two of the authors of this meta-analysis independently extracted all relevant data. Disagreement was resolved by discussion with a third investigator. The following data were extracted: (1) the participants’ demographic data; (2) the lower limb coronal alignment; (3) the coronal alignment of femoral component; (4) the sagittal alignment of femoral component; (5) the blood loss; (6) and the operation time. When data were incomplete or unclear, attempts were made to contact the investigators for clarification. The lower limb coronal alignment was defined as a line bisecting the center of the femoral head, the center of the knee, and the center of the ankle. The coronal alignment of femoral component (which represents varus-valgus angulation) was measured in the coronal plane on the full-limb anteroposterior film. The sagittal alignment of femoral component (which represents flexion-extension angulation) was measured in the sagittal plane on the lateral film [9].

### Statistical methods

This meta-analysis was conducted using RevMan 5.0 (Cochrane Collaboration, Oxford, UK). We assessed the statistical heterogeneity using a standard chi-square test (statistical heterogeneity was considered to be present at *P* < 0.1 and *I* ^2^ values >50%). When comparing trials exhibiting heterogeneity, pooled data were meta-analyzed using a random effects model; otherwise, a fixed effects model was used. Mean differences and 95% confidence intervals (CIs) were calculated for continuous outcomes and risk ratio (RR) and 95% CIs for dichotomous outcomes. Ethical approval was obtained.

## Results

### The included studies

A total of 226 potentially relevant papers were identified. By screening titles and reading the abstracts and entire articles, four studies with 358 knees (178 in the EM group and 180 in the IM group) were included in the final meta-analysis. All of the included studies were RCTs which were level I evidence studies and all published in English. The sample sizes ranged from 50 to 100 knees. The key characteristics of the included RCTs are summarized in Table 1. And the detailed data of the comparison of blood loss between femoral EM and IM technique was showed in Additional file 1.

**Table 1:** Characteristics of the included studies

Author	Country	Patients (EM)/(IM)	Age (EM)/(IM)	Gender (EM)/(IM)	Total knee system	EM system
Jung 2013 [9]	South Korea	56/50	70.4/68.5	Female:male 6:1/5.3:1	PS prosthesis (Stryker)	Mechanical axis marker with IFD measurement
Jeon 2012 [28]	South Korea	40/40	70.1/69.2	Female	PS prosthesis (Stryker)	Markers attached to skin
Baldini 2008 [8]	Italy	50/50	71/70	Female:male 2:1/1.7:1	Posterior stabilized flex fixed-bearing prosthesis (Zimmer)	An extramedullary device with preoperative templated data
Engh 1990 [7]	USA	32/40	69.11(38-88)	Female: 53 Male: 19	Depuy	HDisc–peg taped to skin for intraoperative location

*EM* extramedullary group, *IM* intramedullary group, *IFD* inter-femoral head distance, *ASIS* anterosuperior iliac spine

#### Quality of studies

**Fig. 2:**
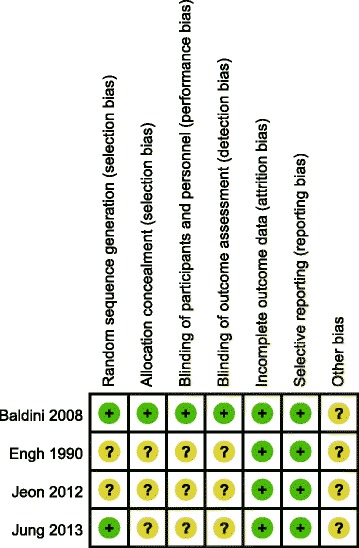
Methodological quality of included studies. This risk of bias tool incorporates assessment of randomization (sequence generation and allocation concealment), blinding (participants, personnel and outcome assessors), completeness of outcome data, selection of outcomes reported, and other sources of bias. The items were scored with “yes”, “no”, or “unsure”

**Fig. 3:**
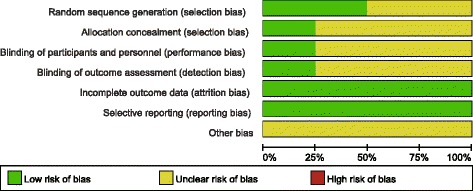
Risk of bias. Each risk of bias item presented as percentages across all included studies which indicated the proportion of different level risk of bias for each item

The methodologic quality of the four included studies was variable. The reported methods of generating allocation sequences were adequate in two studies and one trial reported allocation concealment. Blinding of surgeon and patients were reported in one study and one of the studies blinded their assessors to the outcome. The methodologic quality of the studies is presented in Fig. 2. Judgment with respect to each risk of bias item is presented as a percentage for all of the included studies, as shown in Fig. 3.

### The pooled results of meta-analysis

**Fig. 4:**
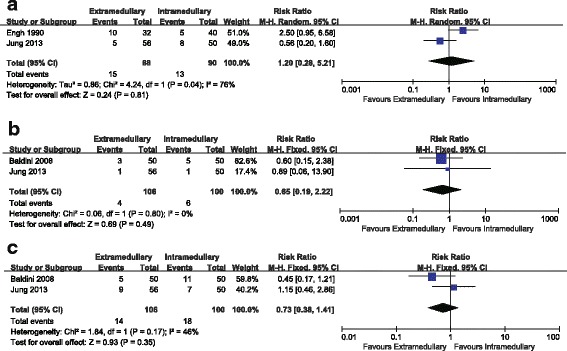
**a** Comparison of the lower limb coronal alignment between femoral EM and IM techniques. **b** Comparison of coronal alignment of femoral component between femoral EM and IM techniques. **c** Comparison of sagittal alignment of femoral component between femoral EM and IM techniques

**Fig. 5:**
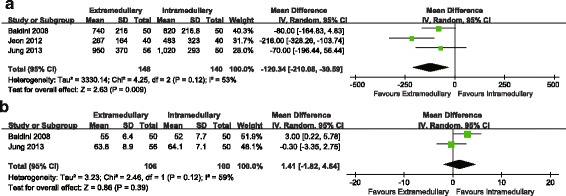
**a** Comparison of blood loss between femoral EM and IM techniques. **b** Comparison of operative time between femoral EM and IM techniques

The pooled results indicated that there was no significant difference between the two groups in terms of the lower limb coronal alignment (RR = 1.20, 95%CI 0.28~5.21, n.s. Fig. 4a). For coronal alignment of femoral component, no significant difference was noted between the two groups (RR = 0.65, 95%CI 0.19~2.22, n.s. Fig. 4b). No significant difference was noted between the two groups in the sagittal alignment of femoral component (RR = 0.73, 95%CI 0.38~1.41, n.s. Fig. 4c). The blood loss was less in the EM group compared with the IM group (MD = −120.34, 95%CI −210.08~−30.59, P =0.009, Fig. 5a). There was no significant difference between the two groups in terms of the operation time (MD = 1.41, 95%CI -1.82~4.64, n.s. Fig. 5b).

## Discussion

Our meta-analysis compared the radiographic outcomes between the EM and the IM femoral guiding technique in patients undergoing TKA. No significant differences were found between the two groups in terms of the lower limb coronal alignment, the coronal alignment of femoral component, and the sagittal alignment of femoral component. The EM guide was associated with a less blood loss and exhibited a similar operation time as the IM guide.

Long-term success after TKA is dependent especially on proper intraoperative component positioning [1–4]. Most authors have reported favorable femoral cuts made with the IM guide and it is considered to be the most accurate with a statistically significant increase in the percentage of distal femoral cuts [6, 7]. Several studies found 85–96% of IM femoral cuts to be acceptable compared with 69–86% of EM cuts. The EM femoral alignment technique had inferior accuracy with approximately 10% more outliers on the coronal plane compared with the IM technique [9]. Historically, all the authors were using the EM instruments referring only to the anterosuperior iliac spine (ASIS) intraoperatively [7, 16]. However, the use of the ASIS to locate the femoral head center (FHC) might not be an accurate method since the FHC was indirectly determined by and dependent on anatomical structures adjacent to the femoral head. Identification of the FHC using the two finger-breadths medial to the ASIS method was unreliable and a wide variation of inter-ASIS distances was found among patients [17].

Recently, some studies have introduced new techniques to improve the accuracy of the extramedullary alignment guide system. Baldini and Adravanti [8] developed a set of EM instruments calibrated with preoperative templating radiograph measurements of inter-femoral head center distance (IFD), which allowed one to perform distal femoral resection without violating the femoral canal. They reported that the femoral component coronal alignment was within 0° ± 2° of the mechanical axis in 84% of the IM group and 86% of the EM group. Matsumoto et al. [18] reported that the femoral component coronal alignment was within 0° ± 3° of the mechanical axis in 98% of patients by using a similar EM instrument. Seo et al. [19] using preoperative templating radiograph measurements of IFD showed that outliers (±3°) of the femoral component coronal alignment were observed in 9.4% of all cases. However, the proximal reference point in the coronal plane was considered to be incorrect when the lower limb was abducted or adducted during surgery. Therefore, Seo et al. [20] reported the extramedullary technique assisted by a mechanical axis marker, which could easily identify the center of femoral head and result in 98.2% of patients achieving acceptable alignment in the range of 0° ± 3° in the coronal plane. Jung et al. [9] reported that the femoral component coronal alignment was within 90° ± 5° in 98.4% of patients using the mechanical axis marker system. This new EM alignment guide system was accurate since it included proximal and distal coronal axis markers to indicate the IFD, which was independent of leg posture. The use of an intramedullary guide for the femur was associated with increased risks of fat embolism, blood loss, postoperative hypoxia, and intraoperative fractures [11, 14, 15]. In addition, the IM femoral alignment system did not always guarantee accuracy of the component position in the TKA. The femoral bowing was a common phenomenon and could affect axial alignment of TKA when IM alignment systems were used, especially in East Asian populations [21, 22]. In these cases, the EM femoral alignment system was a useful alternative surgical option to adjust femoral component alignment [9]. In patients with a more bowed femur, malalignment of lower limb may occur in IM technique, while Computer-assisted techniques could improve the accuracy in both sagittal and coronal planes and EM technique could improve the accuracy in coronal planes. Although the computer-assisted TKA enabled more accurate component alignment [23, 24], the 10-year outcomes of computer-assisted TKA are not superior to that of the conventional technique in function, patient satisfaction and implant survivorship [25, 26].

The opening of the medullary canal using intramedullary jigs was postulated to cause significant blood loss during TKA, although most surgeons have closed the femoral canal opening with bone plug. The application of EM technique was associated with minimal invasiveness since the femoral canal was not breached and the blood loss could be reduced by 145–396 mL [8, 14, 27–29]. Computer-assisted TKA and patient-specific instrumentation (PSI) were recently introduced with the aim of improving alignment without violating the femoral canal [30–32]. TKA using PSI did not result in significantly better femoral component alignment in the sagittal and axial planes than TKA using conventional instrumentation [32]. However, the blood loss was significantly reduced by using the PSI system compared with the IM system for femoral cut. While, the computer-assisted TKA enabled more accurate component alignment. However, it did not reduce the hidden blood loss since the blood loss avoided by not opening the canal might be compensated by greater post tourniquet bleeding due to greater tourniquet time [31].

This present meta-analysis has several limitations. First, only four studies were included and the sample size of the included studies was small, which might have affected our results. Second, we could not perform a valid statistical comparison of the functional outcomes between the two groups. Therefore, further high-quality RCTs with long-term follow-up should be designed to assess radiographic outcomes, knee function, and implant survival rate.

## Conclusions

A satisfactory alignment can be obtained with the use of either intramedullary or extramedullary alignment guide system in TKAs. The use of the extramedullary guide results in less blood loss and exhibits a similar operation time as the intramedullary guide.

## Additional file

Additional file 1: Comparison of blood loss between femoral EM and IM technique (*P* = 0.009). (DOCX 12 kb)

## Abbreviations

CI: Confidence interval
CNKI: China National Knowledge Infrastructure
EM: Extramedullary
IM: Intramedullary
MD: Mean differences
RCTs: Randomized controlled trials
RR: Risk ratio
TKA: Total knee arthroplasty
