# Supplementary material for: Extramedullary versus intramedullary femoral alignment technique in total knee arthroplasty: a meta-analysis of randomized controlled trials
Source: J Orthop Surg Res. 2017 Jun 5;12:82. doi: 10.1186/s13018-017-0582-3 (PMC5460512; doi:10.1186/s13018-017-0582-3)
Supplement: Additional file 1: — Comparison of blood loss between femoral EM and IM technique (P = 0.009). (DOCX 12 kb) [file 13018_2017_582_MOESM1_ESM.docx]

Compasion of blood loss between femoral EM and IM technique （P=0.009）

| Study | Extramedullary | Case | Intramedullary | Case |
| --- | --- | --- | --- | --- |
| Baldini 2008 | 740±216 ml | 50 | 820±216.8ml | 50 |
| Jeon2012 | 267±164ml | 40 | 483±323ml | 40 |
| Jung 2013 | 950±370ml | 56 | 1020±293ml | 50 |
